# Digital Addiction and Related Factors among College Students

**DOI:** 10.3390/healthcare11222943

**Published:** 2023-11-10

**Authors:** Suk-Jung Han, Sugandha Nagduar, Hea-Jin Yu

**Affiliations:** 1 College of Nursing, Sahmyook University, Seoul 01795, Republic of Korea; hansj@syu.ac.kr; 2 Graduate School of Nursing, Sahmyook University, Seoul 01795, Republic of Korea

**Keywords:** digital addiction, university students, body shape satisfaction, time management, psychological heath

## Abstract

(1) Background: Digital addiction has been a global concern, with college students becoming increasingly vulnerable to it. The detrimental psycho-physiological effects of digital addiction have raised concerns regarding college students’ health. This descriptive correlational study was conducted to evaluate the prevalence of digital addiction and identify influencing factors among college students, such as body shape satisfaction, time management, and psychological health. (2) Methods: Data were collected from 199 students at a university in Seoul, South Korea, from 30 May to 13 June 2023. The Digital Addiction Scale (DAS), Time Structure Questionnaire (TSQ), General Health Questionnaire (GHQ), and Body Shape Questionnaire (BSQ) were used for the assessment. Data (N = 199) were analyzed using descriptive statistics, one-way ANOVA, Pearson’s correlation coefficient, and stepwise multiple regression. (3) Results: The mean score for digital addiction was 54 ± 12, with scores ranging from 23.0 to 89.0. Digital addiction had a significant positive correlation with body shape satisfaction (r = 0.156, *p* = 0.028) and a negative correlation with time management (r = −0.500, *p* < 0.001). In the stepwise multiple regression model of digital addiction (adjusted $R^{2}$ = 0.285, *p* < 0.001), subfactors of body shape satisfaction (fear of fatness β = −0.280, body dissatisfaction β = 0.401) and time management (effective organization β = −0.211, persistence β = −0.209, past orientation β = −0.165) were statistically significant. (4) Conclusions: Body shape satisfaction and time management are important factors influencing digital addiction among college students. However, it does not establish a direct causal relationship. The findings indicate that there is a statistical association or connection between these factors, but they do not definitively state that body shape satisfaction or time management directly cause digital addiction. To decrease digital addiction in this population, interventions should aim to improve body shape satisfaction and time management.

## 1. Introduction

In the digital age, social media platforms have become a common means of communication. However, social media addiction is a growing concern, and researchers worldwide are studying its prevalence [1,2]. Digital addiction refers to a harmful dependence on digital media and devices, such as smartphones, video games, and computers. It encompasses various types of addictions, including social networking addiction, smartphone addiction, and internet addiction [3]. Despite this concern, addictive behaviors related to technology are not officially recognized as diagnosable mental health disorders by major diagnostic systems in Western countries. Moreover, there is a lack of consistency in the conceptualization of these phenomena [4]. As of February 2023, South Korea had a 97.6% internet penetration rate and 47.87 million digital media users, representing 92% of the population. On average, South Koreans spend 1 h and 11 min per day on digital media, using 4.5 different platforms. Men and women represent the user base [5]. The term “digital addiction” is often used interchangeably with “internet addiction” [6]. Internet addiction is a behavioral disorder characterized by an overwhelming fixation on internet usage, persistent cognitive patterns centered on self-imposed limitations and regulation of internet use, inability to manage the craving for internet access, persistent engagement with online activities despite negative consequences affecting daily functioning, gradual escalation of time spent online, compulsive drive to seek internet access when unavailable, and unmanageable compulsion to obtain internet connectivity [7,8,9]. The excessive or compulsive use of digital devices, especially among young people, has resulted in numerous adverse outcomes. These include decreased participation in real-life communities, poorer academic performance, relationship issues, negative effects on health related to sleep, attention, and learning, increased risk of obesity and depression, exposure to inaccurate, inappropriate, or unsafe content and contacts, compromised privacy and confidentiality, distraction, reduced social skills, and detrimental effects on psychological or physical well-being [10,11,12]. Studies have revealed that college students’ pursuit of a course, study year, use of internet/day; and the purpose of internet use such as digital networking, games, entertainment, pornography, and news, were significantly associated with internet addiction. Depression and insomnia are correlated with digital addiction [13]. Digital addiction rates are higher in the Eastern Mediterranean region and in low/lower-middle-income countries. Digital addiction varies across regions, economic levels, periods of publication, sex, and assessment scales used in the studies [14]. The high prevalence of internet addiction among junior high school students during the COVID-19 outbreak highlights the need for mental health organizations and educational agencies to develop programs that address and prevent internet addiction among adolescents [15].

Additionally, studies have revealed that awareness and internalization independently mediate the association between SNS addiction symptoms and body-shape dissatisfaction. A significantly higher level of SNS addiction symptoms is linked to increased awareness of appearance pressure, which, in turn, contributes to the internalization of beauty ideals [16]. Emerging evidence suggests a connection between internet use, social media, body image, and eating concerns. The use of the internet, particularly appearance-focused social media, has been linked to increased body image and eating concerns among college students [17]. Problematic usage of the internet (PUI) and its relationship with eating disorders are related to psychological problems. PUI is positively correlated with various aspects of eating disorders and general psychological problems, including overall eating disorder symptoms, body dissatisfaction, drive for thinness, and dietary restraint [18,19,20]. The rise of digital conference platforms has resulted in people constantly checking their appearance and finding flaws in their virtual images. Exposure to social media can exacerbate body image dissatisfaction, fuel social networking site addiction, and worsen the comorbidities of body dysmorphic disorders (BDD) such as depression and eating disorders. Excessive social media use can intensify pre-occupation with imagined image defects among patients with BDD, pushing toward seeking minimally invasive cosmetic and plastic surgery procedures [21]. 

Dysfunctional digital media use affects the time structure of youths. Pre-occupation and fearful attachment patterns are associated with the problematic use of digital media. These attachment patterns also influence self-esteem, fear of missing out (FOMO), and time spent on digital media, fully explaining the link between attachment patterns and problematic digital media use [22,23,24]. Excessive nonacademic internet use negatively affects undergraduate students’ daily lives. Excessive internet use has led to reduced study time and disrupted sleep patterns [25]. Self-control is negatively associated with problematic internet, smartphone, and Facebook use. Furthermore, media multitasking has been identified as a significant mediator in the relationship between self-control and problematic digital media use [23]. The overuse of technology can have negative consequences on an individual’s behavior, emotions, and cognitive abilities [26]. Psychological distress has been identified as a predictor of digital media addiction, and mediating effects of FOMO and boredom proneness have been observed in the relationship between psychological distress and digital media addiction [8,27]. Increased smartphones use and higher digital dependence are associated with lower academic motivation, greater reliance on surface learning, and poorer mental health and quality of life. Digital dependence is a stronger predictor of negative outcomes than smartphone use frequency [28]. 

Influence of idealized body images on digital media platforms has led to body shape dissatisfaction and increase the risk of digital addiction [8]. Time spent on digital devices is positively linked to digital addiction [29]. Digital addiction is associated with psychological health issues like anxiety, depression, and social isolation, as individuals often use digital devices to cope with negative emotions [30]. This study contributes to our understanding of digital media addiction by exploring its prevalence among college students and examining its effects on variables such as body shape dissatisfaction, time spent on digital devices, and psychological health. Focusing on college students reveals a vulnerable population, and the findings may inform the development of prevention and intervention strategies for digital addiction in this group and potentially in other age groups as well.

## 2. Materials and Methods

### 2.1. Study Design and Procedures

This descriptive correlational study assessed the prevalence of digital addiction and its related problems among college students. It was approved by the Institutional Review Board (IRB No. 2023-04-008-001). A total of 200 university students were recruited in Seoul, South Korea, and the final data of 199 students were analyzed. Data were collected from 30 May to 13 June 2023, using convenience sampling. Participants signed an informed consent form. The purpose of the form was fully explained, and an overview of the study was provided. The participants were college students at a university in Seoul, South Korea. The inclusion criteria were university students aged 18 years or older who could read and write Korean. Individuals were requested to complete self-report surveys. Research assistants helped complete the questionnaires as needed. Structured questionnaires were used to assess the knowledge and relative effects of digital media addiction among university students. All participants were remunerated with gift cards to acknowledge the time and effort expended in completing the survey. The tools comprised four scales to address the relative variables of the study: Digital Addiction Scale (DAS), Time Structure Questionnaire (TSQ), General Health Questionnaire (GHQ), and Body Shape Questionnaire (BSQ).

### 2.2. Measures

#### 2.2.1. Outcome Variables

To measure participants’ digital addiction levels and digital device usage, the Digital Addiction Scale (DAS), developed by Kesici and Tunç [31] was used. In study, we used the Korean version of the scale, translated by Kim [32]. The scale consists of 19 questions with 5 subscales (overuse, abstinence, disruption of life flow, emotional state, and dependence), on a 5-point Likert scale, with a total score of 19–95. High scores on this scale indicate higher levels of media addiction. In our study, Cronbach’s alpha was 0.89.

A time-structure questionnaire (TSQ) developed by Feather and Bond [33] was used to measure time management. In this study, we used the Korean version of the scale translated and modified by Jang and Kim [34]. The instrument consists of 26 items and 6 subscales (sense of purpose, structured routine, effective organization, persistence, future orientation, and past orientation) on a 7-point scale, with a total score ranging from 26 to 182. The 26 items are calculated in reverse, with a higher total score indicating better time management skills. In our sample, the internal consistency of the TSQ was excellent (Cronbach’s alpha was 0.96).

A general health questionnaire (GHQ) developed by Goldberg and Williams [35] was used to measure mental health. In this study, we used the Korean version of the instrument translated by Shin [36]. The scale consists of 20 items that measure the risk of mental illness. Higher scores indicate lower levels of mental health. In our sample, Cronbach’s alpha was 0.77.

A body shape questionnaire (BSQ) developed by Cooper et al. [37] was used to measure body shape satisfaction. The BSQ is a 6-point scale with 34 questions; the total score of the BSQ scale ranges from 0 to 34 and consists of four subfactors (fear of obesity, fear of exposure, vomiting, and body shape dissatisfaction), with higher scores indicating lower body satisfaction. In this study, we used the Korean version of the instrument, translated by Noh and Kim [38]. In our sample, Cronbach’s alpha was 0.79.

#### 2.2.2. Sociodemographic Variables

Data on age, sex, major, body mass index (BMI), school year, region, GPA, school life satisfaction, and time spent using digital devices per day were collected through a self-reported survey.

#### 2.2.3. Statistical Analysis

The data were analyzed using SPSS 25 (IBM Corp., Armonk, NY, USA). Demographic characteristics of the participants and study variables were analyzed using descriptive statistics, including frequencies, percentages, means, and standard deviations. One-way ANOVA, Pearson’s correlation coefficient, and stepwise multiple regression tests were used to compare the degree of digital addiction according to the participants’ characteristics.

## 3. Results 

### 3.1. Baseline Characteristics

Baseline characteristics of participants are summarized in Table 1. 

A total of 199 university students participated in the study, of which, 86.8% were aged 19–23 years and 74.4% were female undergraduates. The participants majored in humanities and social sciences (30.2%), health and welfare (29.6%), science and technology (20.6%), future convergence (10.1%), and culture and arts (10.1%). Most participants (69.4%) were first- and second-year university students, and the majority (58.0%) had a BMI range of 18.5 to 22.9. Additionally, 48% were mostly satisfied with school and 32.7% were fairly satisfied with school. Twenty-eight percent of the participants had a GPA of 4.0 to 4.5, 33.9% had a GPA of 3.5 to 4.0, 29.6% had a GPA of 3.0 to 3.5, 6.3% had a GPA of 2.5 to 3.0, and 2.1% had a GPA below 2.5. Furthermore, 68.7% of the participants did not follow a religion.

### 3.2. Participants’ Characteristics Related to Digital Device Usage

As shown in Table 2, the types of digital devices used were TVs (7.2%), smartphones (46.5%), tablets (30.6%), desktop PCs (12.9%), gameplayers (2.3%), and videogames (0.5%). The most frequently used digital devices were smartphones (94%), and the time spent on digital devices per day was less than 5–7 h (25.6%). The most frequent location for digital device use was at home (82.9%), and the most frequent time was from 10 pm to bedtime (49.7%). When analyzing differences in digital addiction according to the general characteristics, female participants had higher scores (54.1 ± 11.3) than male participants (53.0 ± 12.7), and college students majoring in science and technology had the highest scores of all majors (54.5 ± 12.8). Moreover, students without religion had higher digital addiction scores (54.6 ± 11.2) than those with religion (52.0 ± 12.5). Participants with GPAs lower than 2.5 had higher rates of digital addiction (68.3 ± 12.3) than those with higher GPAs. Participants who were hardly satisfied with school life had higher scores (61.3 ± 12.0) than those who were very satisfied (50.3 ± 15.0). Participants who spent more than nine hours per day on digital devices had higher scores than those who spent less than three hours per day on digital devices. 

### 3.3. Descriptive Statistics of the Observed Variables

Table 3 shows the descriptive statistics of the measured variables-the mean score for digital devise usage was 53.8 ± 11.6, with scores ranging from 23 to 89. Looking at the results of the DAS subscale, the highest to lowest average scores were: obsessive use (28.0 ± 5.4) and negative result (25.7 ± 7.7). The mean score for body shape was 91.2 ± 34.1, with scores ranging from 32 to 192. The mean score time structure was 89.5 ± 13.5, with scores ranging from 46 to 118. The mean score for psychological health was 23.7 ± 6.4, with scores ranging from 3 to 45.

### 3.4. Correlations between Research Variables

Table 4, as well as Appendix A and Appendix B, show the Pearson’s correlation (two-tailed) between the variables (Digital addiction, body shape satisfaction, time structure, and psychological health). Digital addiction was significantly positively correlated with body shape satisfaction (r = 0.156, *p* = 0.028) and significantly negatively correlated with time management (r = −0.500, *p* < 0.001). Digital addiction was negatively correlated with psychological health; however, this correlation was not significant (r = −0.019, *p* = 0.794). Moreover, body shape had a significant negative correlation with time structure (r = −0.163, *p* = 0.02) and a significant positive correlation with psychological health (r = 0.232, *p* = 0.001). Finally, time structure was positively correlated with psychological health (r = 0.056, *p* = 0.433).

### 3.5. Factors Affecting Participants’ Digital Device Usage

To determine the effects of body shape satisfaction, time structure, and psychological health on digital addiction, stepwise multiple regression analysis was performed (Table 5). The test for multicollinearity among the independent variables revealed that the tolerance limits for all variables were below 1.0. Additionally, the Durbin–Watson value was 1.914, and the Variance Inflation Factor (VIF) values for all variables were below the threshold of 10, indicating the absence of multicollinearity issues. Furthermore, the homoscedasticity assumption was satisfied, as evidenced by the residual scatter plot, which showed a random distribution around a mean of 0 without exhibiting any specific patterns or trends. Sex, age, and BMI were included as control variables in the regression model. Consequently, the regression model was deemed appropriate. In the finalized stepwise multiple regression model of digital addiction (adjusted R^2^ = 0.285, *p* < 0.001), subfactors of body shape satisfaction (fear of fatness β = −0.280, body dissatisfaction β = 0.401) and time management (effective organization β = −0.211, persistence β = −0.209, past orientation β = −0.165) were statistically significant.

## 4. Discussion

### 4.1. Findings

This descriptive correlational study was conducted to evaluate the prevalence of digital addiction and identify influencing factors among college students, such as body shape satisfaction, time management, and psychological health. It was conducted on 199 undergraduate students to assess the occurrence of digital addiction among students and examine the factors that impact it. It examined the mediating effect of perceived spiritual management on the relationship between job engagement and job satisfaction, and organizational commitment and job satisfaction. 

In our study, we observed differences in digital addiction tendencies based on various baseline characteristics and variables. Firstly, students who reported high satisfaction in their school lives tended to have lower DAS scores compared to those with lower satisfaction. This finding aligns with previous research on teenagers [19,39], suggesting that as digital device usage increases, satisfaction with school life tends to decrease among university students. This decrease in school life satisfaction may lead to a gradual loss of interest in school activities. Consequently, students may become less dedicated to their studies and, simultaneously, invest more time in smartphones, resulting in lower academic performance and reduced satisfaction with their learning activities. Additionally, there is a trend of neglecting social interactions with friends in favor of prioritizing digital device usage among students who experience high digital addiction tendencies.

Furthermore, in our study of baseline characteristics, we observed that students with lower GPAs tended to exhibit higher levels of digital addiction, while students with higher GPAs tended to have lower levels of digital addiction. This result is consistent with findings from previous studies [24,40,41] that have reported significant differences in digital addiction in relation to academic performance. Kolaib, Alhazmi, and Kulaib [42] conducted research on digital addiction among medical students in Saudi Arabia and found that students with lower GPAs tended to have higher instances of digital addiction. Additionally, Qanash et al. [40] conducted a study on 1000 university students majoring in the health sciences to test their level of addiction to electronic devices. It revealed a notable connection between electronic device addiction and decreased GPA. These results highlight the importance of universities implementing targeted interventions to improve academic satisfaction and support, with the aim of mitigating digital addiction. Based on our research findings and previous literatures mentioned above, it is evident that digital media and devices can consume time and attention that should be dedicated to academic pursuits. Excessive use of digital devices can lead to a reduction in the time spent on studying, ultimately having a negative impact on GPA. Therefore, when considering the relationship between low GPA and high digital addiction, it is essential to understand and manage the interaction between academic performance and digital addiction through various approaches. Developing programs and resources that teach students reasonable digital device usage and management habits, support them in focusing on their studies, and help them manage stress is crucial. 

In this study, the subfactors of body shape satisfaction (fear of fatness and body dissatisfaction) influenced digital addiction among undergraduate college students. This result is consistent with previous research that identified the factors influencing internet addiction. Ayran et al. [43] confirmed that body shape satisfaction was significantly associated with internet addiction in Turkey. Moreover, Jang and Kim [34] reported an association between internet addiction and body shape satisfaction among university students. In particular, college students who were highly conscious of their body weight during this period demonstrated that higher obesity levels and body fat percentages were associated with negative body image. Conversely, Kwon et al. [44] found that negative body image increases the likelihood of addiction to digital devices such as smartphones. To address this relationship, it is essential to promote healthy digital usage, improve body shape satisfaction, and address underlying emotional and psychological factors. Encouraging a balanced and mindful approach to digital technology and fostering a positive body image can help mitigate the risks associated with digital addiction. 

In this study, the sub-factors of time management (effective organization, persistence, and past orientation) were significantly positively related to digital addiction, which eventually led to poor academic performance [29,39]. One characteristic of students who spend a lot of time using digital devices is their strong impulsivity and lack of planning/effective orientation in daily life [42]. These findings suggest a strong correlation between digital addiction and time management, indicating that digital addiction can lead to changes in time management. Given these traits, considering impulsive tendencies and the ability to control one’s personal life, there is a pressing need for time management education.

### 4.2. Academic Implications

This research holds academic significance by investigating the factors related to digital addiction among university students, which is a serious issue in today’s world. What sets this research apart is its exploration of a more comprehensive conceptual framework that encompasses the relationships and factors influencing digital addiction among university students. This broader perspective enhances our understanding of the complex dynamics affecting digital addiction, making a valuable scholarly contribution to the field. By studying digital addiction, researchers can develop and evaluate prevention and intervention strategies tailored to university settings. Additionally, academic research can explore how to enhance digital literacy and resilience in university students, helping them navigate the digital world responsibly and mitigate the negative effects of digital addiction.

### 4.3. Practical Implications

In practical terms, we believe that universities can apply these findings to implement concrete guidelines for action in the prevention of digital addictions. Specifically, universities should adopt a comprehensive approach, encouraging students to monitor and manage their screen time. They should also incorporate digital literacy education into the university curriculum to teach students about responsible and effective digital device usage. Establishing peer support groups or mentoring programs where students can connect with peers facing similar challenges is another valuable initiative that universities can undertake to address digital addiction issues.

### 4.4. Limitations

Several limitations of this study warrant further investigation. First, the scope of this study was confined to a single university in South Korea, encompassing a relatively small participant pool. This poses a significant challenge in extrapolating research outcomes to a broader population of university students, necessitating the implementation of multicenter methodologies. Furthermore, the cross-sectional nature of this study may restrict the establishment of causal relationships between the variables. Hence, it is advisable to conduct extensive longitudinal research to effectively address these limitations. 

## 5. Conclusions

This descriptive correlational study assessed the prevalence of digital addiction and its related problems among college students. The specific aim was to explore the factors that contribute to it within the college student population. A group of 200 university students participated in the study, with data from 199 students ultimately subjected to analysis. The overall average score of DAS was 53.5 out of 85. The findings reveal that two crucial factors affecting digital addiction in college students are satisfaction with body shape and effective time management. To mitigate digital addiction among this demographic, interventions should concentrate on enhancing body shape satisfaction and improving time management skills. Additionally, we found that university students with higher scores of DAS tend to have lower GPAs and school life satisfaction. This study holds promise in furnishing empirical groundwork that can inform the development of evidence-based educational and counseling initiatives aimed at preventing digital addiction among college students. Lastly, active efforts by researchers and healthcare professionals are needed to conduct follow-up studies on the prevention of DAS for university students and intervention of the problem of digital addiction in the sample of university students. 

## Figures and Tables

**Table 1:** Baseline Characteristics.

Characteristics	Categories	n	%	Digital Device Usage			
				Mean ± SD	*t* or F	*p*	Scheffė
Gender	Men	51	25.6	53.0 ± 12.7	−0.568	0.570	
	Women	148	74.4	54.1 ± 11.3			
Age * (year) (N = 197)	19	24	12.2	53.7 ± 13.9	1.258	0.284	
	20	64	32.4	55.7 ± 11.4			
	21	42	21.3	53.1 ± 10.4			
	22	21	10.7	49.0 ± 12.7			
	23	20	10.2	56.1 ± 12.7			
	24 or older	26	13.2	53.4 ± 8.9			
Major	Humanities and Social Sciences	60	30.2	54.3 ± 11.7	0.984	0.417	
	Health and Welfare	59	29.6	54.4 ± 10.8			
	Science and Technology	40	20.1	54.5 ± 12.8			
	Future Convergence University	20	10.1	54.0 ± 13.2			
	Culture and Arts	20	10.1	48.9 ± 9.7			
BMI * (N = 193)	<18.5	30	15.5	57.7 ± 15.5	1.302	0.275	
	18.5~22.9	112	58.0	53.2 ± 10.1			
	23~24.9	25	13.0	53.4 ± 12.2			
	≥25.0	26	13.5	53.0 ± 12.6			
School year	First	74	37.2	53.0 ± 10.9	1.544	0.191	
	Second	64	32.2	56.5 ± 12.9			
	Third	33	16.6	51.5 ± 9.7			
	Fourth	26	13.1	51.9 ± 12.6			
	Fifth	2	1.0	57.5 ± 4.9			
Religion * (N = 198)	no	136	68.7	54.6 ± 11.2	1.484	0.140	
	yes	62	31.3	52.0 ± 12.5			
GPA * (N = 189)	4.0 to 4.5	53	28.0	51.4 ± 12.1	2.243	0.066	
	3.5 to less than 4.0	64	33.9	53.4 ± 11.8			
	3.0 to less than 3.5	56	29.6	55.0 ± 11.4			
	2.5 to less than 3.0	12	6.3	54.4 ± 10.7			
	below 2.5	4	2.1	68.3 ± 12.3			
School life satisfaction	hardly satisfied	19	9.5	61.3 ± 12.0 a	3.836	0.011	a > d, a = b, b = c = d
	fairly satisfied	65	32.7	54.5 ± 10.8 b			
	mostly satisfied	93	46.7	52.6 ± 10.7 c			
	very satisfied	22	11.1	50.3 ± 15.0 d			
Time spent on digital devices per day	<3 h a	12	6.0	44.8 ± 10.8 a	11.893	<0.001	a < b < c < e, c = d
	3 to less than 5	50	25.1	49.2 ± 10.1 b			
	5 to less than 7	51	25.6	53.2 ± 10.4 c			
	7 to less than 9	49	24.6	54.2 ± 11.1 d			
	≥9	37	18.6	63.2 ± 10.5 e			

N = 199 * except missing values.

**Table 2:** Participants’ Characteristics Related to Digital Device Usage.

Characteristics	Categories	n	%	% of Total Cases
Used digital devices	TV	31	7.2	15.6
	Smartphone	199	46.5	100.0
	Tablet	131	30.6	65.8
	Desktop, PC	55	12.9	27.6
	Game player	10	2.3	5.0
	Video game	2	0.5	1.0
	Total	428	100.0	215.1
Most frequently used digital device	Smartphones	188	94.5	
	Tablet PC	6	3.0	
	Personal computer	5	2.5	
Time spent on digital device per day	Less than 1 h	2	1.0	
	Less than 1–3 h	10	5.0	
	Less than 3–5 h	50	25.1	
	Less than 5–7 h	51	25.6	
	Less than 7–9 h	49	24.6	
	Less than 9–12 h	30	15.1	
	More than 12 h	7	3.5	
Most frequently used places for digital device use	At home	165	82.9	
	School	8	4.0	
	Public facilities (community centers, government offices, libraries, etc.)	1	0.5	
	While traveling (walking, public transportation, etc.)	15	7.5	
	Use regardless of location	10	5.0	
Time for digital device use	As soon as I wake up (when I wake up)	68	9.6	34.3
	While going to school and returning	112	15.8	56.6
	During class time	31	4.4	15.7
	Mealtime	75	10.6	37.9
	Recess	114	16.1	57.6
	After dinner and before 10 p.m.	90	12.7	45.5
	From 10 p.m. to bedtime	145	20.4	73.2
	Any time	75	10.6	37.9
	Total	710	100.0	358.6
Most frequently used time for digital device use	As soon as I wake up (when I wake up)	4	2.0	
	While going to school and returning	14	7.0	
	During class time	1	0.5	
	Mealtime	1	0.5	
	Recess	20	10.1	
	After dinner and before 10 p.m.	22	11.1	
	From 10 p.m. to bedtime	99	49.7	

**Table 3:** Descriptive Statistics of the Observed Variables.

Variables				Min	Max	Mean ± SD		Item Mean ± SD	
Digital addiction				23.0	89.0	53.8	11.6	2.8	0.6
Obsessive use				11.0	39.0	28.0	5.4	3.5	0.7
Negative result				11.0	52.0	25.7	7.7	2.3	0.7
Body shape satisfaction				32.0	192.0	91.2	34.1	2.9	1.1
Fear of fatness				14.0	84.0	45.4	17.3	3.2	1.2
Shame for exposure of appearance				6.0	36.0	14.1	6.3	2.3	1.1
Purging				2.0	12.0	2.8	1.9	1.4	0.9
Body dissatisfaction				10.0	60.0	28.9	11.5	2.9	1.2
Time management				46.0	118.0	89.5	13.5	4.3	0.6
Sense of purpose				9.0	35.0	21.9	4.8	4.4	1.0
Structured routine				8.0	35.0	21.9	5.0	4.4	1.0
Effective organization				7.0	24.0	16.0	3.2	4.0	0.8
Persistence				6.0	21.0	14.1	3.5	4.7	1.2
Future orientation				2.0	14.0	7.3	2.5	3.6	1.3
Past orientation				2.0	14.0	8.3	2.9	4.1	1.5
Psychological health				3.0	45.0	23.7	6.4	1.2	0.3
Anxiety				0	14	6.2	2.3	1.6	0.6
Depression				0	12	6.2	2.3	1.5	0.6
Social dysfunction				1	50	10.5	4.5	2.6	1.1
Cut-off score	23/24	≤23	95 (47.7)	3	23	18.6	4.0	0.9	0.1
		≥24	104 (52.3)	24	45	28.4	4.1	1.5	0.3
	27/28	≤27	150 (75.4)	3	27	21.1	4.7	1.0	0.1
		≥28	49 (24.6)	28	45	31.8	3.6	1.6	0.1

**Table 4:** Correlations between the Research Variables.

Variables		1	2	3	4
		r (*p*)	r (*p*)	r (*p*)	r (*p*)
1	Digital addiction	1			
2	Body shape satisfaction	0.156 * (0.028)	1		
3	Time structure	−0.500 ** (<0.001)	−0.163 * (0.02)	1	
4	Psychological Health	−0.019 (0.794)	0.232 ** (0.001)	0.056 (0.433)	1

* *p* < 0.05, ** *p* < 0.01.

**Table 5:** Factors affecting Participants’ Digital Device Usage.

Variables	Model 1			
	B	β	*t*	*p*
(constant)	83.946		7.640	0.000
Fear of fatness	−0.191	−0.280	−2.186	0.030
Shame	−0.070	−0.037	−0.267	0.790
Purging	0.045	0.007	0.092	0.927
Dissatisfaction	0.409	0.401	2.441	0.016
Purpose	−0.319	−0.133	−1.704	0.090
Routine	−0.093	−0.039	−0.536	0.592
Organization	−0.770	−0.211	−2.630	0.009
Persistence	−0.707	−0.209	−2.520	0.013
Future	−0.146	−0.031	−0.447	0.656
Past	−0.662	−0.165	−2.010	0.046
Anxiety	−0.731	−0.142	−1.729	0.086
Depression	0.121	0.023	0.291	0.771
Social dysfunction	0.332	0.128	1.782	0.076
Sex	0.061	0.002	0.031	0.975
Age	0.561	0.096	1.482	0.140
BMI	−0.292	−0.080	−1.067	0.287
R^2^	0.345			
Adj. R^2^	0.285			
ΔAdj. R^2^	0.345			
F (*p*)	5.761 (<0.001)			

**Figure A1:**
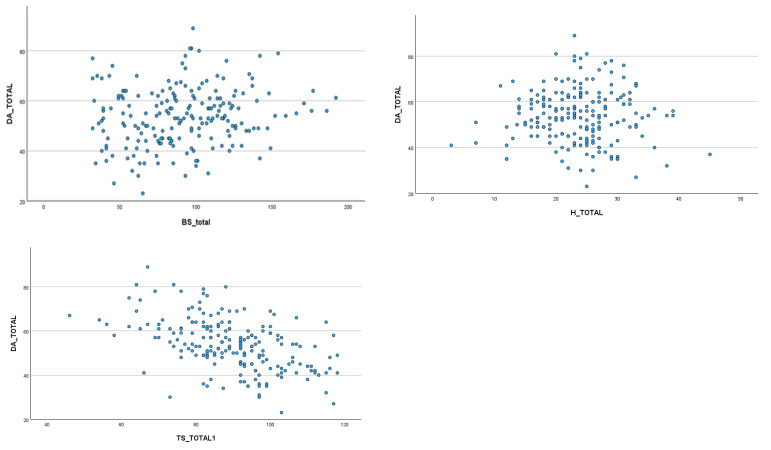
Graph of relations between variables. DA_Total: digital addiction, BS_Total: body satisfaction, TS_Total: time structure (time management), H_Total: psychological health.

## Data Availability

The data presented in this study are available upon request from the corresponding author. The data are not publicly available due to respondents’ privacy.

## Appendix A

**Table A1:** Correlation between the Research Variables.

		1	2	3	4	5	6	7	8	9	10	11	12	13	14	15	16	17	18	19
		r (*p*)	r (*p*)	r (*p*)	r (*p*)	r (*p*)	r (*p*)	r (*p*)	r (*p*)	r (*p*)	r (*p*)	r (*p*)	r (*p*)	r (*p*)	r (*p*)	r (*p*)	r (*p*)	r (*p*)	r (*p*)	r (*p*)
1	Digital addiction	1																		
2	Obsessive use	0.838 ** (*p* < 0.001)	1																	
3	Negative result	0.924 ** (*p* < 0.001)	0.566 ** (*p* < 0.001)	1																
4	Body shape	0.156 * (0.028)	0.109 (0.124)	0.159 * (0.024)	1															
5	Fear of fatness	0.101 (0.157)	0.091 (0.200)	0.088 (0.215)	0.957 ** (*p* < 0.001)	1														
6	Shame for exposure of appearance	0.162 * (0.022)	0.053 (0.459)	0.208 ** (0.003)	0.871 ** (*p* < 0.001)	0.729 ** (*p* < 0.001)	1													
7	Purging	0.074 (0.296)	−0.097 (0.172)	0.181 * (0.011)	0.519 ** (*p* < 0.001)	0.421 ** (*p* < 0.001)	0.522 ** (*p* < 0.001)	1												
8	Body dissatisfaction	0.209 ** (0.003)	0.173 * (0.014)	0.195 ** (0.006)	0.957 ** (*p* < 0.001)	0.859 ** (*p* < 0.001)	0.847 ** (*p* < 0.001)	0.451 ** (*p* < 0.001)	1											
9	Time structure	−0.500 ** (*p* < 0.001)	−0.321 ** (*p* < 0.001)	−0.531 ** (*p* < 0.001)	−0.163 * (0.022)	−0.130 (0.068)	−0.189 ** (0.008)	−0.018 (0.805)	−0.180 * (0.011)	1										
10	Sense of purpose	−0.316 ** (*p* < 0.001)	−0.215 ** (0.002)	−0.327 ** (*p* < 0.001)	−0.192 ** (0.007)	−0.150 * (0.035)	−0.273 ** (*p* < 0.001)	−0.003 (0.970)	−0.191 ** (0.007)	0.711 ** (*p* < 0.001)	1									
11	Structured routine	−0.204 ** (0.004)	−0.023 (0.747)	−0.292 ** (*p* < 0.001)	0.129 (0.070)	0.139 (0.051)	0.077 (0.279)	0.019 (0.786)	0.128 (0.072)	0.603 ** (*p* < 0.001)	0.187 ** (0.008)	1								
12	Effective organization	−0.438 ** (*p* < 0.001)	−0.364 ** (*p* < 0.001)	−0.407 ** (*p* < 0.001)	−0.140 * (0.049)	−0.111 (0.117)	−0.141 * (0.047)	−0.019 (0.79)0	−0.165 * (0.020)	0.681 ** (*p* < 0.001)	0.350 ** (*p* < 0.001)	0.241 ** (*p =* 0.001)	1							
13	Persistence	−0.393 ** (*p* < 0.001)	−0.211 ** (0.003)	−0.446 ** (*p* < 0.001)	−0.156 * (0.028)	−0.162 * (0.023)	−0.106 (0.137)	−0.118 (0.098)	−0.142 * (0.046)	0.708 ** (*p* < 0.001)	0.344 ** (*p* < 0.001)	0.471 ** (*p* < 0.001)	0.427 ** (*p* < 0.001)	1						
14	Future orientation	−0.163 * (0.022)	−0.169 * (0.017)	−0.127 (0.073)	−0.109 (0.124)	−0.117 (0.100)	−0.019 (0.790)	−0.004 (0.956)	−0.137 (0.054)	0.290 ** (*p* < 0.001)	0.107 (0.131)	−0.103 (0.147)	0.213 ** (0.003)	−0.003 (0.968)	1					
15	Past orientation	−0.357 ** (*p* < 0.001)	−0.298 ** (*p* < 0.001)	−0.331 ** (*p* < 0.001)	−0.226 ** (*p =* 0.001)	−0.176 * (0.013)	−0.258 ** (*p* < 0.001)	0.054 (0.448)	−0.271 ** (*p* < 0.001)	0.582 ** (*p* < 0.001)	0.435 ** (*p* < 0.001)	0.029 (0.681)	0.383 ** (*p* < 0.001)	0.244 ** (*p =* 0.001)	0.253 ** (*p* < 0.001)	1				
16	Psychological health	−0.019 (0.794)	−0.040 (0.571)	0.000 (0.999)	0.232 ** (*p =* 0.001)	0.217 ** (0.002)	0.137 (0.054)	0.147 * (0.038)	0.261 ** (*p* < 0.001)	0.056 (0.433)	0.048 (0.502)	0.108 (0.128)	0.086 (0.228)	0.115 (0.107)	−0.152 * (0.032)	−0.105 (0.139)	1			
17	Anxiety	0.033 (0.647)	−0.053 (0.454)	0.087 (0.223)	0.341 ** (*p* < 0.001)	0.291 ** (*p* < 0.001)	0.309 ** (*p* < 0.001)	0.144 * (0.043)	0.376 ** (*p* < 0.001)	−0.106 (0.138)	−0.172 * (0.015)	0.094 (0.188)	0.047 (0.512)	−0.005 (0.939)	−0.148 * (0.038)	−0.281 ** (*p* < 0.001)	0.800 ** (*p* < 0.001)	1		
18	Depression	0.157 * (0.027)	0.144 * (0.042)	0.136 (0.056)	0.335 ** (*p* < 0.001)	0.318 ** (*p* < 0.001)	0.246 ** (*p* < 0.001)	0.058 (0.414)	0.368 ** (*p* < 0.001)	−0.243 ** (*p =* 0.001)	−0.231 ** (*p =* 0.001)	−0.008 (0.912)	−0.056 (0.430)	−0.177 * (0.012)	−0.146 * (0.040)	−0.330 ** (*p* < 0.001)	0.627 ** (*p* < 0.001)	0.564 ** (*p* < 0.001)	1	
19	Social dysfunction	0.011 (0.880)	0.020 (0.777)	0.002 (0.976)	0.010 (0.887)	0.031 (0.665)	−0.109 (0.127)	0.093 (0.192)	0.028 (0.691)	0.165 * (0.020)	0.256 ** (*p* < 0.001)	0.070 (0.324)	0.021 (0.764)	0.153 * (0.031)	−0.161 * (0.024)	0.153 * (0.031)	0.627 ** (*p* < 0.001)	0.248 ** (*p* < 0.001)	0.144 * (0.043)	1

* *p* < 0.05, ** *p* < 0.01.
